# A Population-Based HIV Vaccine Efficacy Trial Design: Statistical Framework and Design Considerations

**DOI:** 10.3390/vaccines14090794

**Published:** 2026-09-09

**Authors:** Holly Janes, Fei Gao, Susan Buchbinder, Charles S. Wiysonge, Kimberly Louis, Blossom Napoleon, Amelia Mfiki, Derrick Mapp, Glenda Gray

**Affiliations:** 1 Vaccine and Infectious Disease Division, Fred Hutchinson Cancer Center, 1100 Fairview Ave N M2-C200, Seattle, WA 98109, USA; fgao@fredhutch.org; 2 Bridge HIV, San Francisco Department of Public Health, University of California, San Francisco, 25 Van Ness Avenue, Suite 100, San Francisco, CA 94114, USA; susan.buchbinder@sfdph.org; 3 Cochrane South Africa, South African Medical Research Council, Francie van Zijl Drive, Parow Valley, Cape Town 7500, South Africa; charles.wiysonge@mrc.ac.za; 4 Vaccine Trials Unit, Fred Hutchinson Cancer Center, 901 Boren Ave, #1320, Seattle, WA 98104, USA; klouis@fredhutch.org; 5 Aurum Klerksdorp Clinical Research Site, Jade Square, OR Tambo Street, Klerksdorp 2570, South Africa; bnapoleon@auruminstitute.org; 6 Groote Schuur Clinical Research Site, South African National Aids Council (SANAC)-Civil Society, J52 Old Main Building Groote Schuur Hospital, Cape Town 8001, South Africa; amelia.mfiki@hiv-research.org.za; 7 Shanti Project, 27 Maiden Lane, Suite 400, San Francisco, CA 94108, USA; dmapp@shanti.org; 8 Infectious Disease and Oncology Research Institute, Faculty of Health Sciences, University of the Witwatersrand, South African Medical Research Council, Tygerberg, P.O. Box 19070, Cape Town 7505, South Africa; glenda.gray@mrc.ac.za

**Keywords:** HIV, vaccines, HIV prevention, bnAbs, clinical trials

## Abstract

An HIV vaccine will be a critical tool for ending the epidemic, yet the next vaccine efficacy trial is likely five or more years away as the field focuses on the early-phase evaluation and optimization of vaccines that induce broadly neutralizing antibodies. At the same time, the HIV prevention landscape has evolved substantially, with the discovery of highly effective means of preventing HIV, challenging the identification of HIV prevention trial designs that are ethically sound and scientifically robust. We explore a population-based, individually randomized, proof-of-concept trial design for evaluating the efficacy of a future candidate vaccine to prevent HIV acquisition and to validate the neutralizing antibody marker as an immune correlate. The design is intended to lay the foundation for a subsequent licensure trial and the development and refinement of future HIV vaccine regimens. Key design features include broad, population-based eligibility criteria; the incorporation of external data; decentralized approaches to recruitment, specimen, and data collection; and strong community partnerships. We compare the approach with alternative trial designs and outline their limitations. The population-based design represents a compelling option for generating credible and generalizable evidence, while enabling future HIV vaccine licensure and development.

## 1. Introduction

The HIV epidemic continues: an estimated 1.3 million people newly acquired HIV globally in 2024, almost unchanged from the prior year [1]. Major disruptions in HIV treatment and prevention in 2025 due to changes in US foreign aid assistance [2] threaten to reverse the reductions in incidence seen over the last decade [3]. Globally, the field is off track to meet the 2030 goals set by UNAIDS targeting the elimination of HIV [1,4]. This is despite dramatic advances in the biomedical prevention of HIV. First oral and now long-acting injectable antiretrovirals used as pre-exposure prophylaxis (PrEP) have been demonstrated to be highly effective for preventing acquisition when used as directed [5,6,7,8,9,10]. Yet there are numerous barriers to the usage of these products including awareness, stigma, cost, logistics, and cultural, geographic, socio-economic, behavioral and lifestyle factors [11]. Even when available, access may be limited to pregnant and/or breastfeeding women or key populations most impacted by HIV—sex workers, men who have sex with men, transgender women, and people who inject drugs and their sex partners—and require individuals to self-identify as vulnerable to HIV which further limits initiation and continued use in settings where these specific features drive stigma. Data suggest that most adolescents, pregnant women, and young adults acquiring HIV did not perceive themselves to be especially vulnerable [12,13,14,15]. Moreover, globally, an estimated 45% of new HIV acquisitions occur outside of key populations [16]. The figure is even higher in high-incidence regions including eastern and southern Africa where an estimated 77% of new HIV acquisitions occur outside the populations targeted for PrEP services. A low-cost vaccine, even one with modest efficacy, has the potential to reduce HIV incidence sustainably and cost-effectively [17,18,19]. Ending the HIV epidemic will ultimately require an effective HIV vaccine in addition to other highly effective, readily available and affordable HIV prevention and treatment strategies [20,21].

The HIV vaccine field has investigated numerous approaches, beginning with the VAX003 and VAX004 trials of a recombinant HIV envelope gp120 protein subunit vaccine that demonstrated that antibodies alone—in particular non-neutralizing strain-specific antibodies—were insufficient for protection [22,23]. The STEP and Phambili trials of the MRKAd5 HIV-1 Gag/Pol/Nef vaccine demonstrated that T cells with limited breadth could not prevent acquisition [24,25]. Then came the turning point: in 2009, the RV144 trial in Thailand provided the first proof-of-concept that an HIV vaccine could work, with the ALVAC-HIV (vCP1521) prime and recombinant gp120 protein AIDSVAX B/E boost conferring a modest but significant 31.2% efficacy; the high initial efficacy of 60% waned over 12 months [26,27] and failed to replicate when studied in a different population, with a different adjuvant, and redesigned for a Clade C epidemic [28].

Two foundational studies have paved the path forward for HIV vaccine development. First, the RV144 trial demonstrated that IgG antibodies targeting the V1V2 loop of gp120 correlated with the level of protection; Fc-mediated effector functions, especially Antibody-Dependent Cellular Cytotoxicity (ADCC) in the absence of Env-specific IgA antibodies, as well as CD4+ T cells also contributed, but protection waned rapidly [29,30]. Second, the AMP studies (HVTN 703/HPTN 081, HVTN 704/HPTN 085), which evaluated the passively infused VRC01 antibody, provided proof of concept that broadly neutralizing antibodies (bnAbs) can prevent HIV acquisition [31] and demonstrated that serum neutralizing antibodies predict the level of protection [32], establishing quantitative benchmarks for a bnAb-inducing vaccine. Given that most circulating viruses detected in the AMP trials were found to be resistant to VRC01, the data suggest that a single bnAb is not enough; combination bnAbs that target different epitopes on the HIV envelope likely will be needed, either induced by active vaccination or passive infusion. Informed by these insights, numerous vaccine strategies are under investigation that seek to induce HIV bnAbs. Lineage-based vaccines and germline-targeting vaccines are advancing from pre-clinical phases to phase 1 clinical trials, conducted domestically and internationally, to evaluate vaccine safety and the induction of precursors of bnAbs targeting conserved epitopes on the HIV envelope [29,33]. Different vaccine dosing and delivery strategies are being investigated, including fractionated and bolus delivery, slow-release formulations, and novel adjuvants. Given that these bnAb vaccines are in the discovery phase, an efficacy trial is likely five or more years in the future.

When a candidate bnAb-inducing HIV vaccine regimen is ready for efficacy evaluation, designing the efficacy trial is likely to be challenging because of the existence of highly effective modalities to prevent HIV acquisition. Nonetheless, the burden of HIV persists in certain geographical areas and populations; determining how to evaluate the preventive efficacy of a candidate vaccine in this context is of critical importance. The field of antiretroviral-based PrEP has also wrestled with efficacy trial design challenges [34,35,36,37]. However, trial designs that are appropriate for evaluating new PrEP agents are arguably different from those for a vaccine, in part because an HIV vaccine may serve populations that are not served by PrEP, including those outside key populations who constitute most new HIV acquisitions in many regions around the world [16]. In addition, an HIV vaccine first entering a proof-of-concept trial is unlikely to be as effective as long-acting injectable PrEP.

Another distinctive feature of the HIV vaccine field is the emerging consensus around a likely correlate of protection for a bnAb-inducing HIV vaccine. Evidence from the HIV field suggests that the vaccine-induced neutralizing antibody response against circulating strains of HIV is likely to be a strong correlate of protection [32]. This is further supported by non-human primate challenge studies [38,39,40,41] and broader experience in vaccinology, where neutralizing antibodies often serve as correlates of protection and the basis for vaccine modification and regulatory approval [42,43,44,45]. Accordingly, the magnitude and breadth of the neutralizing antibody response to relevant circulating HIV strains is highly likely to form a central basis for go/no-go decisions on advancing a vaccine candidate to an efficacy trial. The next efficacy trial must be designed to validate the neutralizing antibody response as an immune correlate to support the further refinement of the vaccine and, ultimately, the regulatory approval of future vaccines.

This manuscript will consider and evaluate a specific design for a potential HIV vaccine efficacy trial: a large, population-based trial, similar in concept to the Salk inactivated polio vaccine trial of 1954 [46]. We describe the trial design and the motivations behind it, evaluate its operating characteristics, and discuss the challenges with the design and additional potential design features. We compare the population-based trial design to alternative trial designs that might be considered, including those that have been used for evaluating new PrEP agents, carefully articulating their limitations for the HIV vaccine context. We conclude with a discussion of the steps the field must take to lay the groundwork for the next HIV vaccine efficacy trial.

## 2. A Population-Based, Individually Randomized, Placebo-Controlled Trial Design

We explore a leading potential HIV vaccine efficacy trial design that is developed under the following premises. Our first premise is that this trial will be a proof-of-concept trial that evaluates whether the vaccine protects individuals from acquiring HIV. However, it is likely that the vaccine regimen that is tested will require modification before it can be licensed and used in public health practice. It may be that the number of vaccine doses or the mode of delivery must be simplified, the adjuvant needs replacing, or that multiple immunogens are needed to increase the breadth of protection. The trial will be pivotal for establishing proof of concept, but will not be the primary basis for vaccine licensure. Thus, we have designed the trial using a phase 2b design [47], a design that is large enough to evaluate the preventive efficacy of the vaccine regimen, but not to determine whether it is efficacious enough for public health impact [47,48]. Our second premise is that the phase 2b trial will only be conducted following phase 1 and 2a studies of the vaccine regimen, wherein these earlier studies will confirm that the vaccine regimen is safe, well-tolerated, and generates immune responses plausibly predicting efficacy in preventing HIV acquisition. In particular, a predicted vaccine efficacy of at least 50% will likely be needed to initiate the phase 2b trial. Finally, we assume that the phase 2b trial will need to validate the neutralizing antibody marker as an immune correlate of risk (CoR) for this vaccine and provide strong support that it is also likely a correlate of protection (CoP). A CoR is a biomarker that is associated with HIV acquisition among vaccine recipients, whereas a CoP is a biomarker that predicts the level of vaccine efficacy [49,50] such as the neutralizing antibody titer, which is established as a CoP for a wide array of COVID-19 vaccines [51,52]. This evidence of an immune correlate will provide a critical foundation for the HIV vaccine field: it will support and inform the modification of the vaccine regimen leading up to a phase 3 trial and enable decision-making around future HIV vaccines and their efficacy in new populations [44,53]. We envision that a vaccine modified such that the predicted vaccine efficacy is 70% or higher, based on the neutralizing antibody response and a prediction model built using the phase 2b trial data, would support the launch of a phase 3 trial.

A future vaccine efficacy trial will need to address three primary objectives: 1. evaluate vaccine safety and tolerability; 2. evaluate vaccine efficacy to prevent HIV acquisition; and 3. validate the neutralizing antibody marker as an immune CoR. Key secondary objectives are to evaluate the neutralizing antibody marker as a CoP and to estimate a model of vaccine efficacy as a function of the marker to enable informed decision-making around improved versions of this vaccine or related vaccines. Importantly, vaccine efficacy must be measured relative to a placebo, to establish proof of concept that the vaccine works, noting that the vaccine and placebo are administered on top of a background standard of prevention. In contrast, establishing the efficacy of the vaccine relative to a proven intervention, e.g., injectable PrEP, is not compelling as it is not anticipated that the vaccine regimen entering the phase 2b trial will be as effective as injectable PrEP, and the vaccine is not an alternative intervention to injectable PrEP. Moreover, establishing vaccine efficacy relative to PrEP would leave considerable uncertainty about the level of efficacy of the vaccine, and this uncertainty would be a barrier to the future development of the vaccine regimen. Section 7 discusses additional ethical and clinical challenges with a study design that would instead evaluate the relative efficacy of the vaccine vs. PrEP.

**Figure 1:**
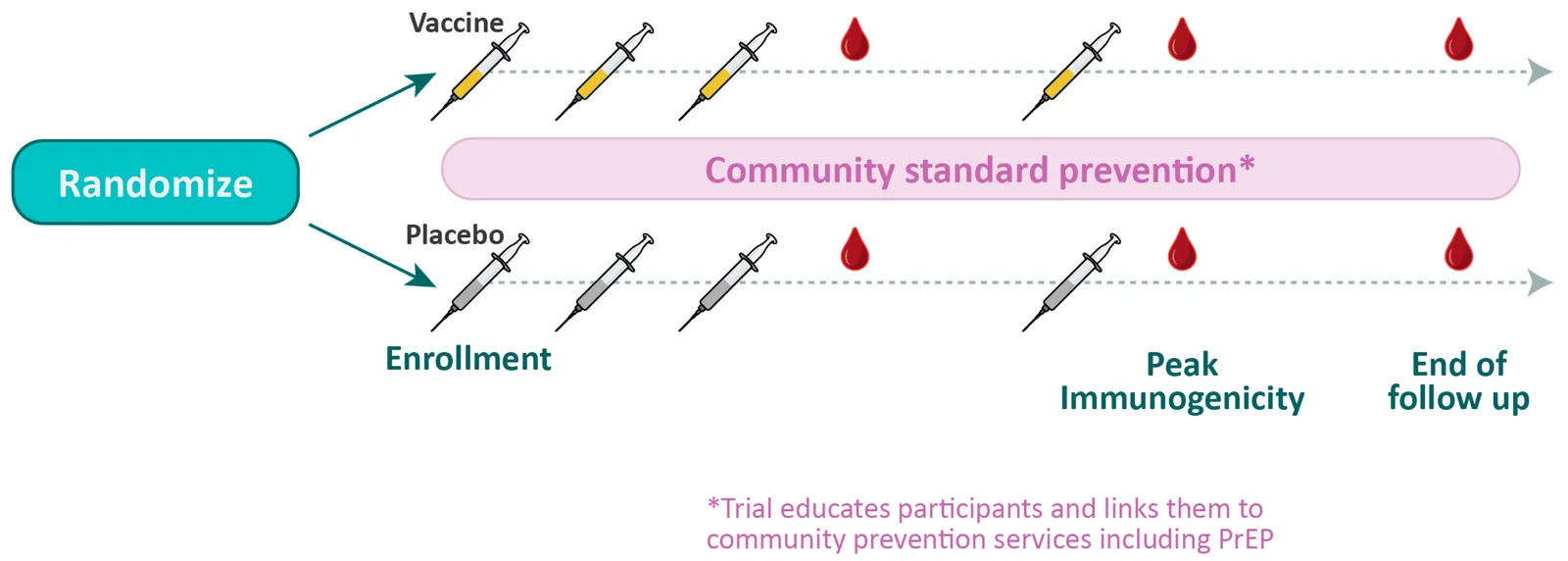
Schema of a population-based, individually randomized, placebo-controlled HIV vaccine efficacy trial. Participants are referred to or provided access to community-based standard prevention throughout follow-up, including linkage to community PrEP services.

For concreteness, we consider designing an HIV vaccine efficacy trial among young women (18–35 years old) in southern Africa, where there is one of the highest burdens of HIV globally [1]. The population-based design enrolls young women vulnerable to acquiring HIV in communities with high HIV burden as depicted by antenatal seroprevalence surveys and routine HIV testing services. Women in subpopulations with targeted HIV prevention strategies implemented by their Ministries of Health, e.g., sex workers, are not enrolled. Rather, the design enrolls young women vulnerable to acquiring HIV by virtue of living in areas with high HIV prevalence. Eligibility criteria are as broad as possible, to enhance recruitment and generalizability. At screening, enrollment, and throughout the trial, women are educated about effective prevention of HIV and linked to PrEP and other HIV prevention services in the community; thus, it is important that the trial is conducted in areas in which some form of PrEP is available. These two design elements—recruiting young women from the general population and leveraging the community standard of prevention, defined in partnership with the community—are aspects of the population-based design that differentiate it from a traditional HIV prevention efficacy trial (Table 1). Young women enrolling in the trial are randomized to receive the vaccine or a placebo, where all trial participants have access to community-based HIV prevention modalities (Figure 1). The design answers what is arguably the most critical and community-relevant question around an HIV vaccine: Can the vaccine reduce HIV incidence further, beyond what has been achieved with the currently available HIV prevention package? Blinding of trial participants to the intervention, i.e., the use of a placebo, is critical for reliably evaluating the ability of the vaccine to protect. Prior experience in HIV [54] and other infectious disease trials [55] has demonstrated that knowledge of the intervention received may affect participant behavior and threaten the reliable estimation of vaccine efficacy. Additionally, experience demonstrates that in an open-label trial, participant retention and compliance with study visits and procedures are likely to differ across arms [56], thus affecting the capture of HIV acquisition and safety outcomes.

To enable the enrollment of a large number of young women, study activities—including study visits, procedures, specimen collections, and data collections—are as simple as possible. Study interventions are limited to the delivery of the vaccination series, which may occur over four or five visits, and blood is collected at enrollment, at the putative peak immunogenicity time point—often two weeks after the final vaccination—and at the end of study follow-up. For concreteness, we consider a vaccine regimen that takes one year to deliver and assume all participants are followed for safety and regular HIV testing for an additional year following the last vaccination, using community-based and remote study procedures as discussed below. Notably, many of the bnAb-inducing vaccine regimens under evaluation require multiple doses to be administered to guide the immune system toward bnAb induction. Participants who acquire HIV during the course of the trial are linked to HIV care, potentially including viral resistance testing and first-line ART regimen selection in the context of long-acting injectable PrEP, and are followed for at least 6 months to ensure treatment is initiated and to evaluate potential post-HIV-acquisition vaccine effects.

The population-based trial design embraces informed personal choice, i.e., different people will prefer different means of HIV prevention, and their choices are dynamic and influenced by an array of contextual factors including an expanding set of prevention options [57,58]. The HIV prevention products available and the manner of access to them may vary across geographic regions and trial sites, over the course of the trial, and over time for individual trial participants, and this heterogeneity aids answering the scientific question.

## 3. Sample Size and Trial Operating Characteristics of the Population-Based Design

The statistical power of the HIV vaccine trial design depends on the number of participants who acquire HIV over the course of the trial. Table 2 shows the number of HIV acquisitions to ensure 90% power to reject the null hypothesis that vaccine efficacy (VE) is below 0% under an assumed alternative VE. Here, VE is measured as 1 minus the ratio of HIV acquisitions in the vaccine vs. placebo groups and is assumed to vary between 50% and 70%; a moderate VE is reasonable to assume for a vaccine regimen first entering proof-of-concept testing. We focus on a design in which young women are randomized 2:1 to vaccine: placebo because unequal randomization increases power for studying immune correlates. Power for correlates analysis primarily depends on the number of HIV acquisitions that accrue in the vaccine arm [50,59]. To ensure adequate power to validate the neutralizing antibody marker as a CoR, the sample size calculations also require that the trial accrue at least 30 HIV acquisitions in the vaccine group under the alternative VE; this benchmark has been used as a guide for powering studies for immune correlates in advance of information about the properties of markers to be studied, although strong immune correlates have been discovered with fewer cases [60,61]. Note that if the alternative VE is below 55%, the number of HIV acquisitions is driven by the primary objective to evaluate VE, whereas for a higher alternative VE, the number is driven by the primary CoR objective and at this point the number of HIV acquisitions increases with higher alternative VE (Table 2). To detect 60% vaccine efficacy, 68 HIV acquisitions provide 90% power and the expected vaccine:placebo acquisition split is 31:37—remembering that this corresponds to VE = 60% given the 2:1 randomization ratio.

The total sample size of the trial depends on the number of HIV acquisitions for 90% power, as well as the underlying HIV incidence in the population (Table 3). HIV incidence among young women in recent HIV prevention efficacy trials in southern Africa has been approximately 3–4% annually [62]. We vary the assumed HIV incidence between 0.75% and 2.0% to be conservative, allowing for declining HIV incidence at a population level and the enrollment of young women outside populations targeted for HIV prevention. We also allow for a conservative 15% annual loss to follow-up (LTFU), approximately 50–100% higher than that seen in recent trials among young women in southern Africa [8,28,31,63] given the enrollment of a population-based cohort that may have a lower retention rate. Finally, we assume that the primary VE and immune correlates analyses count acquisitions that accrue two or more weeks after the final vaccination to allow vaccine-induced immunity to mount, and we assume that follow-up continues through 24 months for all participants. Thus, the design is a hybrid of an event-driven design and a fixed follow-up design; the sample size is selected to ensure that sufficient events accrue, but all participants are followed for 24 months for maximal information.

Continuing to follow participants once sufficient events have accrued maximizes the contribution of each participant and the resources invested in standing up the trial and is also advantageous for evaluating how VE varies over time and as a function of the immune response. As shown in Table 3, 10,067 participants are required to detect 60% vaccine efficacy assuming a 1.5% placebo-group HIV incidence.

**Figure 2:**
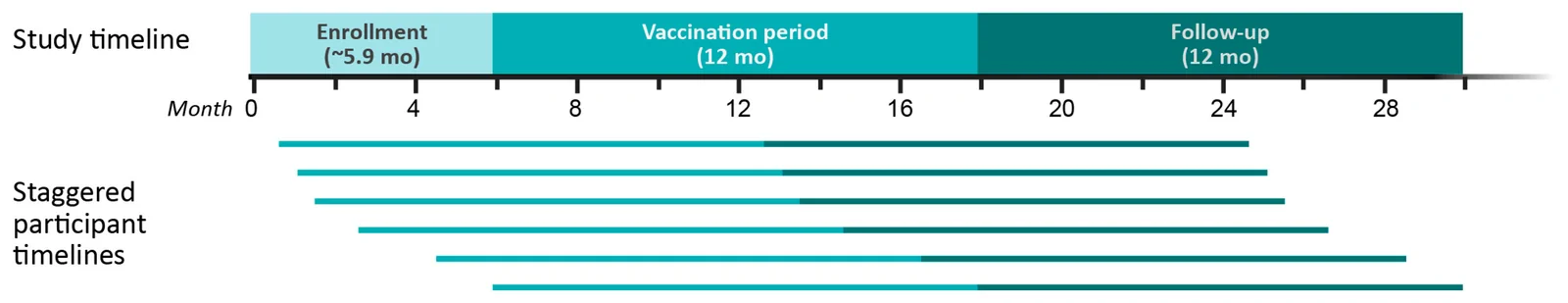
Timeline for a trial enrolling 10,067 participants at a rate of 1700 per month, designed for 90% power to detect 60% VE, rejecting H_0_: VE ≤ 0% using a 1-sided 0.025-level log-rank test, 2:1 randomization vaccine:placebo, requiring at least 30 HIV infections expected in the vaccine group under the alternative VE to ensure adequate power for immune correlates analyses, and assuming 1.5% placebo-group HIV and 15% annual loss-to-follow-up. Assumes a 1-year vaccination series and 24 months of follow-up per participant. An expected 5.9 months of enrollment and total trial duration of 29.9 months are expected.

The total duration of the trial depends on the sample size and the enrollment rate (Table 4). A trial enrolling 10,067 participants to detect 60% vaccine efficacy with 90% power under a 1.5% placebo-group HIV incidence would take 5.9 months to enroll and extend 29.9 months in total, assuming an enrollment rate of 1700 participants per month, e.g., 34 participants per site per month with 50 sites, or 85 per month with 20 sites (Figure 2). Note that the primary driver of trial duration in this context is the duration of the vaccine regimen; here, we assume it extends over 1 year. A shortened vaccine regimen would reduce trial duration and likely enhance participant retention.

The COVID-19 pandemic demonstrated the feasibility of rapidly enrolling large numbers of participants in vaccine efficacy trials, albeit in the context of a global pandemic. Trials of 14,000–50,000 participants were enrolled in several months with estimated enrollment rates of 30–130 participants per site per month in South Africa [64,65,66,67,68]. Decentralized recruitment strategies, discussed below, may enhance recruitment [69,70].

## 4. Anticipated Challenge: Uncertain HIV Incidence

A major challenge with the population-based design is the uncertainty surrounding the population HIV incidence rate, especially in the context of a dynamic and expanding HIV prevention portfolio. A trial that accrues too few HIV acquisitions risks being underpowered. To address this, the design is event-driven: the sample size is selected to safeguard that the number of HIV acquisitions ensuring 90% power is accrued over the course of the study. Dried blood spots could be collected in a subset of trial participants, with real-time testing for ARVs used to provide an early read on the usage of PrEP in the trial population. In addition, interim monitoring of HIV incidence in the trial population is conducted to allow the sample size to be expanded if early evidence suggests that HIV incidence is lower than anticipated.

Lessons learned around oral PrEP and its impact on HIV incidence have some relevance. Evidence of oral PrEP efficacy was first established in 2010, and daily dosing was recommended for people at substantial HIV risk by the World Health Organization in 2015 [71]. Tenofovir disoproxil fumarate/emtricitabine (TDF-FTC) was licensed as oral PrEP in South Africa in 2015 and introduced to the public sector in 2016; annual HIV incidence among 15–59-year-olds declined by 45–73% across regions of South Africa over the following decade [72], which may have been largely due to the roll-out of treatment preventing transmissions. The dapivirine vaginal ring, licensed in South Africa in 2022, has been found to have lower rates of uptake among adolescents and young women relative to oral PrEP [73,74]. Despite these advances, HIV incidence remains unacceptably high, at 3–4% annually among young women in placebo groups of recent HIV prevention efficacy trials in southern Africa [62]. Oral PrEP demonstration projects–even while conducted among populations with a stated interest in PrEP–have found low rates of PrEP persistence [75,76,77]. Numerous lines of evidence point to a variety of socio-behavioral, cultural, and economic factors influencing the levels of oral PrEP uptake and persistence [78,79].

The extent to which the lessons around oral and ring PrEP apply to injectable PrEP is unclear. In theory, long-acting injectable PrEP should address the challenges in terms of inadequate persistence with oral PrEP which requires frequent dosing. However, injectable PrEP has different implementation challenges including injection site pain for cabotegravir and common, visible, and long-lasting injection-site nodules for lenacapavir [80], and the high cost of injectable PrEP may prohibit widespread access, especially in resource-limited settings. Generic versions of cabotegravir and lenacapavir could become available in low- and middle-income countries as soon as 2027. As injectable PrEP becomes more accessible, lessons will be learned about its real-world use. Key questions are: What is short- and long-term adherence? How do different populations embrace it? And how effective is injectable PrEP as drug levels wane? This last question is critical for understanding HIV incidence in populations with varying adherence to injectable PrEP. The extraordinarily high efficacy of PrEP in the context of clinical trials has posed challenges to understanding how efficacy varies as a function of drug levels. At the same time, PrEP regimens will continue to evolve, likely becoming less expensive and employing dosing strategies that address barriers to use. Understanding the uptake of these regimens, their persistence patterns, and their efficacy as a function of adherence, will be critical to refining the trial design.

An additional feature of the population-based design could be considered to further mitigate the risk posed by uncertain HIV incidence. Advances in statistical methodology support pursuing secondary analyses of vaccine efficacy that augment the trial data with external control data [81,82] such as recency tests among those screened for trial participation and historical or concurrent observational HIV incidence data applicable to the target population. Importantly, these augmented analyses are best viewed as secondary analyses, i.e., they would support the interpretation of primary vaccine efficacy analyses that are imprecise if HIV incidence is unexpectedly low in the trial population.

**Figure 3:**
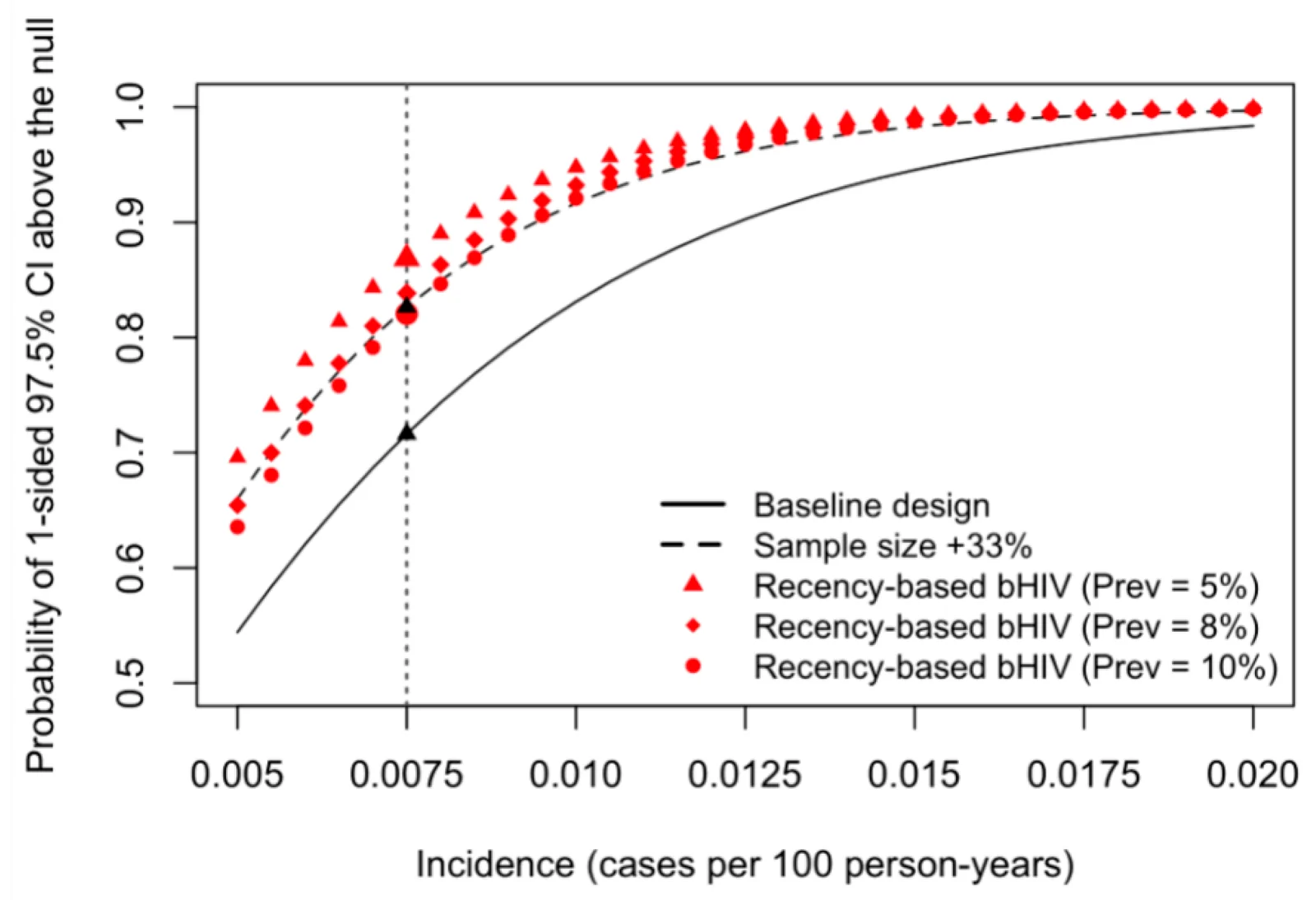
Probability that a one-sided 97.5% confidence interval for VE excludes VE = 0% based on analyses that augment the population-based design with external control data, as a function of the placebo-group HIV incidence. The black curve is the probability for the “baseline” population-based design that enrolls 10,067 participants to detect 60% vaccine efficacy with 90% power under 2:1 randomization and 1.5% placebo incidence. The red curves represent the probability for the baseline design that also applies HIV recency testing to all individuals screened for trial participation, assuming 5%, 8%, or 10% HIV prevalence among screened individuals. The black dashed curve is a reference and shows the probability for a design that is one-third larger than the baseline design: it enrolls 13,390 participants who are randomized 1:1 to vaccine:placebo. All calculations assume a one-year vaccination series, a 15% annual loss to follow-up, and that the primary analysis counts infections 2 weeks post-last-immunization through month 24.

To illustrate this concept, suppose, for example, that HIV recency tests are given to all individuals screened for the population-based trial. With the recency testing data, the HIV incidence in the population from which participants are recruited can be estimated [83,84,85]. Consider the population-based design with 10,067 participants to detect 60% vaccine efficacy with 90% power under a 1.5% placebo-group HIV incidence. The power to evaluate VE is 94.5%, exceeding the target of 90% but additionally satisfying the requirement of at least 30 acquisitions in the vaccine group for immune correlates analysis. This design will be underpowered if incidence is in fact 0.75%, i.e., 50% lower than anticipated. Figure 3 demonstrates this by considering the properties of confidence intervals at the end of the trial, whereas power is relevant in advance of the trial. For the 10,067-person trial, the probability that a one-sided 97.5% confidence interval excludes the null of VE = 0% is 71.6% if the placebo-group incidence is 0.75%. In contrast, this probability is 82.1%, 83.8%, or 86.9% if the trial is augmented with HIV recency data for those screened for trial participation, assuming 10%, 8%, or 5% of those screened are positive for HIV and recency test performance as assumed for the Purpose 1 trial in adolescent girls and young women [10] (recent acquisition if the normalized optical density from the Sedia Limiting Antigen Avidity Enzyme Immunoassay (LAg-EIA) is below 1.5 and HIV-1 RNA viral load > 1000 copies/mL; mean duration of recent acquisition = 184 days, relative standard error = 7%; false recency rate = 1.5%, relative standard error = 70%; 70% of those HIV-negative at screening enroll). For comparison, the probability is 82.6% for a much more resource-intensive approach of expanding the trial sample size by one-third, for a total of 13,390 participants. Notably, these calculations assume that the external control provides an unbiased estimate of placebo HIV incidence, and thus its intended use in the population-based design is to enhance secondary, rather than primary, estimates of vaccine efficacy. In summary, augmentation with external control data is a resource-efficient approach to increasing the precision of vaccine efficacy estimation which is especially important if trial incidence is lower than expected.

## 5. Optimizing Compliance and Recruitment

Decentralization, e.g., using mobile vans, buses, and temporary “pop-up” clinics to deliver the vaccine, is a key attribute of the population-based design (Table 1). Widely implemented for routine immunization programs and supplementary immunization activities in low-resource and geographically dispersed settings, decentralized approaches have consistently demonstrated their value in reaching populations that are otherwise missed by facility-based services, including rural communities, informal urban settlements, mobile populations, and individuals facing structural barriers such as transport costs or time constraints [86,87]. Applying these strategies to an HIV vaccine efficacy trial is anticipated to substantially reduce participant burden by bringing trial services closer to where people live and work, thereby improving recruitment and enhancing the representativeness of the study population. Importantly, these approaches can also foster community trust and engagement when implemented in partnership with local leaders and health workers, and trust is especially critical for an HIV vaccine trial where stigma and hesitancy may affect trial participation.

Experience from outreach vaccination campaigns also highlights the operational feasibility of delivering complex interventions outside traditional clinical settings, provided that systems are carefully designed [88,89]. Mobile teams have successfully administered vaccines, conducted screening, and collected biological specimens in field conditions, often supported by standardized protocols, task-shifting to trained mid-level providers, and strong supervisory structures. For the population-based HIV vaccine trial, this could suggest a hybrid model wherein initial screening and consent are conducted at the community level, with mobile units facilitating follow-up visits, specimen collection, and data collection and adverse event monitoring. Digital tools—such as electronic data capture, real-time monitoring dashboards, and mobile communication platforms—can further strengthen decentralization by enabling timely data transmission, participant tracking, and adaptive management of field operations. Self-collection of HIV status using self-testing or self-collection of blood (e.g., using the Tasso devices) that are mailed and tested in a central lab may provide additional resolution on the timing of HIV acquisition, which is important for immune correlates analyses. Central lab testing is preferred for vaccines that may cause vaccine-induced seroreactivity (VISR)- a determination that will be made in prior phase 1 and 2 studies of the vaccine; the central laboratory will use a specialized diagnostic algorithm to differentiate HIV infection from VISR. While VISR rates have been low in recent phase 1 trials of candidate bnAb-inducing vaccines (J. Hural, personal communication, 26 August 2026), VISR likely needs to be entirely ruled out in order for home testing to be employed. The HIV diagnostic algorithm that is employed will also need evaluation in the context of the evolving state of knowledge around accurate HIV infection diagnosis in the context of long-acting injectable PrEP [90].

However, decentralization introduces important risks that must be proactively mitigated. Maintaining cold-chain integrity for investigational products is more complex in mobile settings and requires robust solutions, including validated portable cold-chain equipment, continuous temperature monitoring devices, and contingency plans for equipment failure. Similarly, ensuring data quality and protocol adherence across dispersed sites necessitates rigorous standard operating procedures, comprehensive staff training, and frequent quality assurance checks, potentially supported by remote monitoring technologies. Another consideration is the standard practice for HIV prevention trials to provide comprehensive sexual health care to trial participants—including contraceptive counseling and screening for STIs. Mobile services could be designed to incorporate these services as well. Participant retention, often a challenge in decentralized trials [91,92], can be addressed through strategies proven in immunization programs, such as appointment reminders, community-based follow-up, and flexible scheduling aligned with participants’ daily routines. The population-based design may also have lower compliance with study procedures than a trial conducted in a high-incidence subpopulation due to competing life challenges. The sample size calculations above anticipate this: a substantially lower rate of retention is used than that seen in recent HIV prevention efficacy trials among young women in southern Africa. Secondary analyses must be prospectively planned to address potential bias caused by lower retention rates for specific populations (i.e., informative censoring) and to evaluate vaccine efficacy in a hypothetical world in which all individuals receive the full vaccination series. Overall, while mobile and outreach approaches introduce logistical complexity, the experience from immunization programs suggests that, when well-executed, they can substantially enhance equity, feasibility, and external validity in large-scale vaccine trials [69,86,92].

Adherence to the vaccine regimen may be a challenge for a bnAb-inducing vaccine regimen that likely requires four or more doses to protect. Large, population-based vaccine trials most often evaluate simpler vaccine regimens, and many take place when there is already evidence of vaccine efficacy based on prior randomized trials. Attention to minimizing the number of vaccine doses will be needed to maximize adherence in the efficacy trial.

Given that the population-based design seeks to enroll young women in the general population, screen-to-enroll rates may differ from those seen in previous HIV prevention trials conducted in high-risk subpopulations. At the same time, adopting community-based recruitment practices used by outreach vaccination campaigns, including through community health clinics, community outreach programs, and using mobile clinics, is expected to increase the reach of recruitment strategies. Employing broad eligibility criteria obviates the need for risk-based screening practices common in traditional HIV prevention trials in high-risk subpopulations, thus enhancing the efficiency of screening. These features of the population-based design support the feasibility of enrolling large numbers of young women in the general population, while enhancing the generalizability of trial results.

## 6. Community Partnership: A Necessity of a Population-Based Design

Community engagement will be essential for trial success, which depends on dedicated participation, community trust, and alignment with community priorities [93,94]. Sustained community advisement throughout all stages of the design, implementation, and dissemination process will be essential. Early and substantive engagement with diverse community representatives to evaluate this design relative to other options discussed below will ensure that the trial design concept is feasible and responsive to local concerns. Incorporating community input early in development and continuously throughout the trial design process will foster transparency between the researchers and the community, address potential misconceptions, and foster mutual learning and shared ownership of the research. Community partnership is part of the stakeholder engagement needed in advance of the trial, including with regulatory partners.

The trial’s approach to PrEP is a critical component of this population-based concept, and community, regulatory, and ethics input will be essential. The proposed approach is to leverage community services to implement HIV prevention and for the study to educate and link participants to these services. However, through community partnerships, it will be important to evaluate the perceptions of PrEP and barriers to it, e.g., based on stigma or social harms. Previous HIV prevention efficacy trials have, in some instances, covered the costs of PrEP and delivered it to participants to reduce financial and logistical barriers (see e.g., [95]), or offered PrEP to participants after completion of the trial (e.g., [8]). These strategies do not align with the concept of a population-based design; the provision of PrEP does not reflect the real-world standard of prevention, which likely varies across regions, and such provision changes the question the trial is answering. Furthermore, the provision of PrEP may reduce trial HIV incidence to levels at which the study objectives cannot be addressed. This issue will require considerable community advisement to ensure alignment.

The language used to describe the trial design must be explored through community partnerships. In particular, it is essential that participants clearly understand what a placebo is, why it is used, and that all participants are offered access to all available HIV prevention modalities, including long-acting agents, throughout trial follow-up. Information about the placebo must be provided in the local language and framed using locally meaningful terms or analogies to support understanding [96]. Providing clear and transparent information about the use of a placebo is critical for building trust and maintaining the integrity of the trial.

Ongoing community engagement throughout trial implementation will be essential for recruitment and retention efforts, as community members will be able to advise on strategies for maximizing both. Community-led information sessions will be critical to allow potential trial participants to engage on the specifics of the study design and will be crucial in supporting dissemination of trial results.

## 7. Alternative Trial Designs and Their Limitations

A variety of additional trial designs have been put forward in the HIV prevention field, including trial designs used to evaluate the efficacy of new PrEP products. Table 5 lists these alternative designs and their key limitations relative to the population-based design. The limitations of the alternative designs fall into several distinct areas. First, several alternative designs face greater challenges in terms of feasibility. For example, a population-based, cluster-randomized trial that randomizes clinics or geographical regions to deliver the HIV vaccine or a placebo would share all the challenges of the individually randomized, population-based design. In addition, it would require a larger sample size by virtue of the within-cluster correlation and reduced vaccine efficacy that is typical of cluster-randomized designs [97]. Another alternative design, an individually randomized trial in a population of unmet need (similar to the MOSAICO design used to study the efficacy of the Ad26.Mos4.HIV vaccine [98]), incorporates additional uncertainty in recruitment speed and feasibility, given the need to specifically recruit individuals who choose not to take up existing HIV prevention modalities despite linkage to low/no-cost options. While such an ‘enrichment’ approach may work for some pathogens with defined high-attack-rate populations [99], it is challenging to use for HIV, for which there may exist highly effective means of prevention. The population-based design does not exclude participants using existing means of HIV prevention, e.g., oral, topical, or injectable PrEP.

Another recurring theme among the alternative designs is the presence of ethical concerns related to equipoise and differential access to effective HIV prevention. While the population-based design provides access to effective prevention to all trial participants, an active-controlled design, such as the HPTN 083 and HPTN 084 double-blind, double-dummy designs used to establish the efficacy of long-acting cabotegravir [7,8], would randomize trial participants to the HIV vaccine or a proven HIV prevention modality as active control, e.g., oral or injectable PrEP. Thus, under an active-controlled, double-blind double-dummy design, only a portion of trial participants—those in the active control arm—would receive access to prevention known to be effective. Not only does this pose ethical questions, but it also makes the clinical management of trial participants challenging if the trial is blinded; clinicians and participants will not know if they are already receiving effective prevention. Therefore, the vaccine-arm participants will not have access to PrEP, should they desire it, because they will not know if they are already receiving PrEP. Equipoise may also be in question when randomizing participants to a vaccine first entering proof-of-concept testing vs. injectable PrEP. Operationalizing the trial in a double-blind fashion, i.e., with placebo versions of both the vaccine and the active control, would create complex and burdensome clinic visits and procedures and would limit trial participation to those who are willing to receive both a vaccine and PrEP. In practice, the target population for a vaccine may be those not willing to take or not currently using PrEP.

Reduced reliability of trial results is another limitation of several alternative designs. An active-controlled trial may be augmented with external control data used to estimate a ‘counterfactual placebo’ HIV incidence, as was done for the Purpose 1 and Purpose 2 trials establishing the efficacy of injectable lenacapavir for HIV prevention [9,10]. The primary analysis would compare HIV incidence in the vaccine arm with the counterfactual placebo HIV incidence. This approach suffers from the same ethical considerations as the active-controlled trial previously described and is less rigorous and less convincing to the community and regulators than an internally randomized control group; bias in the counterfactual placebo HIV incidence estimate may result in an incorrect conclusion around the level of vaccine efficacy. While this is not a major threat for an injectable PrEP agent for which there is considerable pre-trial evidence supporting plausible high-level efficacy, and for which the observed HIV incidence in the injectable PrEP arm is nearly zero, this is a major issue for an early vaccine prototype anticipated to have modest efficacy. The impact of bias in the counterfactual placebo is much greater for a product with modest efficacy. As illustrated above, while a counterfactual placebo may improve precision in secondary vaccine efficacy analyses, its use as the basis for primary efficacy evaluation entails considerable risk. An even more extreme design, a single-arm trial in which participants all receive the candidate HIV vaccine, would be much less credible to the community and regulators than a trial with an internally randomized control.

## 8. Future Directions

Much preparatory work is needed to enable a future efficacy trial of a bnAb-inducing HIV vaccine. Beyond optimizing the regimen for high preventive efficacy, it will be essential to simplify the vaccination schedule and ensure high tolerability—requirements that are central to the feasibility of a population-based trial design. Investments in these areas will not only strengthen trial implementation but also facilitate the eventual translation of an effective HIV vaccine into public health practice. Furthering knowledge of vaccine immune responses and potential correlates of vaccine efficacy will support the rational downselection and qualification of regimens for efficacy testing, while also informing the design of immune correlate analyses in the efficacy trial. Adapting and evaluating decentralized approaches for the population-based design will necessitate targeted pilot testing, stress-testing of decentralized outcome ascertainment and follow-up systems, and logistical and infrastructure analyses. In parallel, deepening our understanding of the barriers to and facilitators of HIV vaccine uptake, alongside existing prevention modalities, is critical to ensure that the trial is conducted in the appropriate population and that prevention strategies reflected in the study design are informed by community needs. Collecting and synthesizing data around the uptake, persistence patterns, and efficacy as a function of adherence to contemporary PrEP regimens will sharpen assumptions about HIV incidence that undergird the trial design. Early and sustained community, regulatory, and ethical engagement will be essential to achieving alignment on trial design and implementation.

Vaccine development is a long and resource-intensive process. Recent work has highlighted several strategies that may be employed to shorten the path to licensure for investigational vaccines [99]. While many of the derisking strategies described—such as human challenge studies and reliance on non-human efficacy data—are generally not applicable to HIV vaccines given the limited predictive value of preclinical systems, the concept of leveraging an established immune correlate of protection to advance vaccine development does apply. The neutralizing antibody marker, while not yet a validated correlate of protection for an HIV vaccine, is highly likely to perform well as a surrogate outcome, based on evidence from the HIV field and the broader vaccine field. The current challenge is that investigative HIV vaccines do not yet generate levels of broadly neutralizing antibodies that would be predicted to confer high protection from acquisition. And yet, having the neutralizing antibody marker as an arbiter of candidate vaccines and delivery strategies serves to focus the field and rationally refine and select vaccines for further testing. As the vaccines advance, the neutralizing antibody marker can form the basis for informed go/no-go decisions at each phase of research, with levels predicting at least 50% vaccine efficacy likely needed to support launching a phase 2b trial. It is critical to ensure that the phase 2b trial is large enough to validate the neutralizing antibody marker as a correlate of risk and a likely correlate of protection, thus enabling a phase 3 design to be justified for a vaccine that induces high enough levels of this biomarker to predict 70% efficacy or more. In addition, the strategic overlap of phase 1, 2a, 2b, and 3 evaluations of an HIV vaccine, with prespecified go/no-go decisions built in at each stage, may shorten the development pathway, while limiting opportunities for iterative reassessment and requiring advancement decisions reliant on criteria defined in advance of complete safety, immunogenicity, and proof-of-concept data.

## 9. Conclusions

A population-based, individually randomized trial design represents a compelling option for a future phase 2b trial of a candidate bnAb-inducing HIV vaccine. Anticipated challenges, including uncertainty in HIV incidence and participant adherence and retention, can be addressed through a combination of complementary strategies, of which decentralized trial design features are an important component. With these elements in place, the population-based design is well-positioned to generate results that are credible and persuasive, while also generating the data needed to validate the neutralizing antibody marker as an immune correlate, thereby laying the foundation for eventual HIV vaccine licensure and future HIV vaccine development.

## Figures and Tables

**Table 1:** Attributes of a population-based HIV vaccine trial in comparison to a traditional HIV vaccine trial.

	Population-Based Design	Traditional Design	Implications for Population-Based Design
Trial population	General population, with broad eligibility criteria in communities with high HIV burden	High-risk subpopulation, e.g., in key population(s) or with multiple behavioral risk factors for HIV	Results of a population-based trial generalize to the general population instead of high-risk subpopulation(s)
Background standard of prevention provided to all trial participants	Referral to community-based HIV prevention services	Trial standard of prevention, i.e., prevention “package” provided that may cover the cost of PrEP or prescribe/deliver PrEP	Vaccine efficacy is interpretable as the percentage reduction in HIV incidence, relative to the community standard of prevention instead of the trial standard of prevention
Participant recruitment centers	Community health clinics, community outreach programs, mobile clinics or social media	Clinical research sites or social media	Recruitment of more diverse and representative participants in the community
Intervention delivery and specimen collection	Decentralized via community health clinics, mobile clinics, and self-collection	At clinical research site	Trial procedures are easier for participants to access, which may enhance retention and adherence

**Table 2:** Number of HIV acquisitions for 90% power to detect the specified level of vaccine efficacy (VE), rejecting H_0_: VE ≤ 0% using a 1-sided 0.025-level log-rank test, 2:1 randomization vaccine:placebo, and with at least 30 HIV acquisitions expected in the vaccine group under the alternative VE to ensure adequate power for immune correlates.

	VE = 50%	VE = 55%	VE = 60%	VE = 65%	VE = 70%
No. HIV acquisitions (vaccine:placebo)	99 (50:49)	75 (36:39)	68 (31:37)	73 (31:42)	80 (30:50)

**Table 3:** Total trial sample size for 90% power to detect the specified level of vaccine efficacy (VE), rejecting H_0_: VE ≤ 0% using a 1-sided 0.025-level log-rank test, 2:1 randomization vaccine:placebo, and with at least 30 HIV infections expected in the vaccine group under the alternative VE to ensure adequate power for immune correlates analyses. Calculations assume a 1-year vaccination series, 15% annual loss-to-follow-up, and that the primary analysis counts infections 2 weeks post-last-immunization through Month 24.

Annual Placebo-Group HIV Incidence	VE = 50%	VE = 55%	VE = 60%	VE = 65%	VE = 70%
0.75%	26,111	20,822	19,927	22,650	26,374
1.0%	19,652	15,671	14,997	17,046	19,849
1.5%	13,193	10,520	10,067	11,443	13,325
2.0%	9964	7945	7603	8642	10,063

**Table 4:** Expected duration of trial enrolling 10,067 participants, designed for 90% power to detect 60% VE, rejecting H_0_: VE ≤ 0% using a 1-sided 0.025-level log-rank test, 2:1 randomization vaccine:placebo, requiring at least 30 HIV infections expected in the vaccine group under the alternative VE to ensure adequate power for immune correlates analyses, and assuming 1.5% placebo-group HIV incidence. Assumes 1 year vaccination series, 15% annual loss-to-follow-up, and that primary analysis counts infections 2 weeks post-last-immunization through Month 24.

Enrollment Rate (Participants/Month)	Enrollment Duration (Months)	Trial Duration (Months)
1400	7.7	31.7
1700	5.9	29.9
2000	5.0	29.0
2300	4.4	28.4
2600	3.9	27.9

**Table 5:** Alternative potential study designs for a future HIV vaccine efficacy trial and their key limitations.

Alternative Trial Design	Key Limitations Relative to Population-Based, Individually Randomized Design
1. Population-based, cluster-randomized trial	Larger than an individually randomized design, by virtue of within-cluster correlation With few clusters, there is a risk of imbalance in cluster characteristics between arms and potential confounding A smaller vaccine effect size is expected
2. Placebo-controlled RCT in population with unmet need, i.e., MOSAICO-like design enrolling only those who actively decline PrEP despite having access to low/no cost PrEP.	The size of the population with unmet need who can be recruited to the trial is uncertain; the trial may become infeasible as the population becomes small or difficult to identify and recruit The study provision of highly effective prevention may be necessary, thus reducing HIV incidence to a level at which objectives cannot be assessed Study results may not generalize to the population served by the vaccine
3. Active-controlled, double blind double dummy trial, e.g., HPTN 083/084 design	A potential lack of equipoise allowing randomization to the vaccine vs. highly effective prevention Only some study participants–those in the control arm–receive highly effective prevention. Participants in the vaccine arm are unable to use highly effective prevention, even if they desire it A complicated study schedule to administer both products in double-blind fashion If the active control is a moderately effective prevention method (e.g., oral PrEP in young African women), highly effective prevention must still be available. Clinical management is challenging under double-blinded design, i.e., participants and clinicians do not know if they are already receiving effective prevention.
4. Active-controlled trial augmented with counterfactual placebo, e.g., Purpose 1&2 designs	Limitations of Design 3, in addition to: The efficacy of the vaccine relative to a counterfactual placebo is less reliable given the comparison of non-randomized groups; this is especially true for a vaccine with modest efficacy
5. Single-arm trial with counterfactual placebo	Unlikely to be acceptable to regulators or the scientific community High risk: no ‘plan B’ in the event that the counterfactual placebo yields unexpectedly low or high incidence

## Data Availability

No new data were created or analyzed in this study. Data sharing is not applicable to this article.

## References

[1] UNAIDS 2024 Global AIDS Report—The Urgency of Now: AIDS at a Crossroads. https://www.unaids.org/en/resources/documents/2024/global-aids-update-2024.

[2] Kates J., Rouw A., Oum S.U.S. Foreign Aid Freeze & Dissolution of USAID: Timeline of Events. https://www.kff.org/global-health-policy/u-s-foreign-aid-freeze-dissolution-of-usaid-timeline-of-events/.

[3] Ratevosian J., Beyrer C. (2026). Fundamental PEPFAR Reform Risks a Period of Structural Vulnerability in the HIV Response. Lancet.

[4] (2025). Global Task Team on 2030 HIV Targets Writing Group. Global HIV Targets: A Roadmap to 2030 and Beyond. Lancet.

[5] Grant R.M., Lama J.R., Anderson P.L., McMahan V., Liu A.Y., Vargas L., Goicochea P., Casapía M., Guanira-Carranza J.V., Ramirez-Cardich M.E. (2010). Preexposure Chemoprophylaxis for HIV Prevention in Men Who Have Sex with Men. N. Engl. J. Med..

[6] Baeten J.M., Donnell D., Ndase P., Mugo N.R., Campbell J.D., Wangisi J., Tappero J.W., Bukusi E.A., Cohen C.R., Katabira E. (2012). Antiretroviral Prophylaxis for HIV Prevention in Heterosexual Men and Women. N. Engl. J. Med..

[7] Landovitz R.J., Donnell D., Clement M.E., Hanscom B., Cottle L., Coelho L., Cabello R., Chariyalertsak S., Dunne E.F., Frank I. (2021). Cabotegravir for HIV Prevention in Cisgender Men and Transgender Women. N. Engl. J. Med..

[8] Delany-Moretlwe S., Hughes J.P., Bock P., Ouma S.G., Hunidzarira P., Kalonji D., Kayange N., Makhema J., Mandima P., Mathew C. (2022). Cabotegravir for the Prevention of HIV-1 in Women: Results from HPTN 084, a Phase 3, Randomised Clinical Trial. Lancet.

[9] Kelley C.F., Acevedo-Quiñones M., Agwu A.L., Avihingsanon A., Benson P., Blumenthal J., Brinson C., Brites C., Cahn P., Cantos V.D. (2025). Twice-Yearly Lenacapavir for HIV Prevention in Men and Gender-Diverse Persons. N. Engl. J. Med..

[10] Bekker L.-G., Das M., Karim Q.A., Ahmed K., Batting J., Brumskine W., Gill K., Harkoo I., Jaggernath M., Kigozi G. (2024). Twice-Yearly Lenacapavir or Daily F/TAF for HIV Prevention in Cisgender Women. N. Engl. J. Med..

[11] Ngcobo S.J., Zhandire T. (2025). Pre-Exposure Prophylaxis Uptake, Implementation and Barriers in Africa: A Scoping Review Protocol. Int. J. Environ. Res. Public Health.

[12] Heck C.J., Reed D.M., Okal J., Chipeta E., Mbizvo M., Mathur S. (2024). Examining Concordance of Sexual-Related Factors and PrEP Eligibility with HIV Risk Perception among Adolescent Girls and Young Women: Cross-Sectional Insights from DREAMS Sites in Kenya, Malawi, and Zambia. BMC Public Health.

[13] Slaymaker E., Calvert C., Marston M., Risher K., Imai-Eaton J.W., Moorhouse L., Price A., Abdul R., Dube A., Nabukalu D. (2026). Individual and Population-Level Risk Factors for New HIV Infections among Adults in Eastern and Southern Africa. Nat. Commun..

[14] Torres T.S., Teixeira S.L.M., Hoagland B., Konda K.A., Derrico M., Moreira R.I., Guanira J.V., Benedetti M., Nazer S., Calvo G.M. (2023). Recent HIV Infection and Annualized HIV Incidence Rates among Sexual and Gender Minorities in Brazil and Peru (ImPrEP Seroincidence Study): A Cross-Sectional, Multicenter Study. Lancet Reg. Health Am..

[15] Lancki N., Almirol E., Alon L., McNulty M., Schneider J.A. (2018). Preexposure Prophylaxis Guidelines Have Low Sensitivity for Identifying Seroconverters in a Sample of Young Black MSM in Chicago. AIDS.

[16] Korenromp E.L., Sabin K., Stover J., Brown T., Johnson L.F., Martin-Hughes R., Brink D.T., Teng Y., Stevens O., Silhol R. (2024). New HIV Infections Among Key Populations and Their Partners in 2010 and 2022, by World Region: A Multisources Estimation. JAIDS J. Acquir. Immune Defic. Syndr..

[17] Adamson B., Garrison L., Barnabas R.V., Carlson J.J., Kublin J., Dimitrov D. (2019). Competing Biomedical HIV Prevention Strategies: Potential Cost-effectiveness of HIV Vaccines and PrEP in Seattle, WA. J. Int. AIDS Soc..

[18] Adamson B., Dimitrov D., Devine B., Barnabas R. (2017). The Potential Cost-Effectiveness of HIV Vaccines: A Systematic Review. PharmacoEconomics Open.

[19] Harmon T.M., Fisher K.A., McGlynn M.G., Stover J., Warren M.J., Teng Y., Näveke A. (2016). Exploring the Potential Health Impact and Cost-Effectiveness of AIDS Vaccine within a Comprehensive HIV/AIDS Response in Low- and Middle-Income Countries. PLoS ONE.

[20] Lehmann C., Schommers P. (2024). The Need for Novel Approaches to HIV-1 Vaccine Development. Lancet Infect. Dis..

[21] Jatt L.P., Mgodi N.M., Buchbinder S.P., Gray G.E., Kublin J.G. (2025). An HIV Vaccine in the Era of Twice-Yearly Lenacapavir for PrEP—Essential or Irrelevant?. N. Engl. J. Med..

[22] Flynn N.M., Forthal D.N., Harro C.D., Judson F.N., Mayer K.H., Para M.F., rgp120 HIV Vaccine Study Group (2005). Placebo-Controlled Phase 3 Trial of a Recombinant Glycoprotein 120 Vaccine to Prevent HIV-1 Infection. J. Infect. Dis..

[23] Pitisuttithum P., Gilbert P., Gurwith M., Heyward W., Martin M., van Griensven F., Hu D., Tappero J.W., Choopanya K., Bangkok Vaccine Evaluation Group (2006). Randomized, Double-Blind, Placebo-Controlled Efficacy Trial of a Bivalent Recombinant Glycoprotein 120 HIV-1 Vaccine among Injection Drug Users in Bangkok, Thailand. J. Infect. Dis..

[24] Gray G.E., Allen M., Moodie Z., Churchyard G., Bekker L.-G., Nchabeleng M., Mlisana K., Metch B., de Bruyn G., Latka M.H. (2011). Safety and Efficacy of the HVTN 503/Phambili Study of a Clade-B-Based HIV-1 Vaccine in South Africa: A Double-Blind, Randomised, Placebo-Controlled Test-of-Concept Phase 2b Study. Lancet Infect. Dis..

[25] Buchbinder S.P., Mehrotra D.V., Duerr A., Fitzgerald D.W., Mogg R., Li D., Gilbert P.B., Lama J.R., Marmor M., del Rio C. (2008). Efficacy Assessment of a Cell-Mediated Immunity HIV-1 Vaccine (the Step Study): A Double-Blind, Randomised, Placebo-Controlled, Test-of-Concept Trial. Lancet.

[26] Robb M.L., Rerks-Ngarm S., Nitayaphan S., Pitisuttithum P., Kaewkungwal J., Kunasol P., Khamboonruang C., Thongcharoen P., Morgan P., Benenson M. (2012). Risk Behaviour and Time as Covariates for Efficacy of the HIV Vaccine Regimen ALVAC-HIV (vCP1521) and AIDSVAX B/E: A Post-Hoc Analysis of the Thai Phase 3 Efficacy Trial RV 144. Lancet Infect. Dis..

[27] Rerks-Ngarm S., Pitisuttithum P., Nitayaphan S., Kaewkungwal J., Chiu J., Paris R., Premsri N., Namwat C., de Souza M., Adams E. (2009). Vaccination with ALVAC and AIDSVAX to Prevent HIV-1 Infection in Thailand. N. Engl. J. Med..

[28] Gray G.E., Bekker L.-G., Laher F., Malahleha M., Allen M., Moodie Z., Grunenberg N., Huang Y., Grove D., Prigmore B. (2021). Vaccine Efficacy of ALVAC-HIV and Bivalent Subtype C Gp120–MF59 in Adults. N. Engl. J. Med..

[29] Haynes B.F., Saunders K.O., Hahn B.H., Wiehe K., Baden L.R., Shaw G.M. (2025). Status of HIV Vaccine Development: Progress and Promise. J. Int. AIDS Soc..

[30] Kim J.H., Excler J.-L., Michael N.L. (2015). Lessons from the RV144 Thai Phase III HIV-1 Vaccine Trial and the Search for Correlates of Protection. Annu. Rev. Med..

[31] Corey L., Gilbert P.B., Juraska M., Montefiori D.C., Morris L., Karuna S.T., Edupuganti S., Mgodi N.M., deCamp A.C., Rudnicki E. (2021). Two Randomized Trials of Neutralizing Antibodies to Prevent HIV-1 Acquisition. N. Engl. J. Med..

[32] Gilbert P.B., Huang Y., deCamp A.C., Karuna S., Zhang Y., Magaret C.A., Giorgi E.E., Korber B., Edlefsen P.T., Rossenkhan R. (2022). Neutralization Titer Biomarker for Antibody-Mediated Prevention of HIV-1 Acquisition. Nat. Med..

[33] Stamatatos L. (2025). The Germline Targeting Vaccine Concept: Overview and Updates from HIV Pre-Clinical and Clinical Trials. Curr. HIV Res..

[34] Donnell D., Kansiime S., Glidden D.V., Luedtke A., Gilbert P.B., Gao F., Janes H. (2024). Study Design Approaches for Future Active-Controlled HIV Prevention Trials. Stat. Commun. Infect. Dis..

[35] Gao F., Janes H., Buchbinder S., Donnell D. (2025). Active-Controlled Trial Design for HIV Prevention Trials with a Counterfactual Placebo. Stat. Med..

[36] Cutrell A., Donnell D., Dunn D.T., Glidden D.V., Grobler A., Hanscom B., Stancil B.S., Meyer R.D., Wang R., Cuffe R.L. (2017). HIV Prevention Trial Design in an Era of Effective Pre-Exposure Prophylaxis. HIV Clin. Trials.

[37] Glidden D.V., Stirrup O.T., Dunn D.T. (2020). A Bayesian Averted Infection Framework for PrEP Trials with Low Numbers of HIV Infections: Application to the Results of the DISCOVER Trial. Lancet HIV.

[38] Pegu A., Borate B., Huang Y., Pauthner M.G., Hessell A.J., Julg B., Doria-Rose N.A., Schmidt S.D., Carpp L.N., Cully M.D. (2019). A Meta-Analysis of Passive Immunization Studies Shows That Serum-Neutralizing Antibody Titer Associates with Protection against SHIV Challenge. Cell Host Microbe.

[39] Pauthner M.G., Nkolola J.P., Havenar-Daughton C., Murrell B., Reiss S.M., Bastidas R., Prévost J., Nedellec R., von Bredow B., Abbink P. (2019). Vaccine-Induced Protection from Homologous Tier 2 SHIV Challenge in Nonhuman Primates Depends on Serum-Neutralizing Antibody Titers. Immunity.

[40] Richel E., Cordsmeier A., Bauer L., Fraedrich K., Vestweber R., Roshani B., Stolte-Leeb N., Ensser A., Stahl-Hennig C., Überla K. (2024). Mechanisms of Sterilizing Immunity Provided by an HIV-1 Neutralizing Antibody against Mucosal Infection. PLoS Pathog..

[41] Julg B., Sok D., Schmidt S.D., Abbink P., Newman R.M., Broge T., Linde C., Nkolola J., Le K., Su D. (2017). Protective Efficacy of Broadly Neutralizing Antibodies with Incomplete Neutralization Activity against Simian-Human Immunodeficiency Virus in Rhesus Monkeys. J. Virol..

[42] Klasse P.J., Moore J.P. (2022). Reappraising the Value of HIV-1 Vaccine Correlates of Protection Analyses. J. Virol..

[43] Doria-Rose N.A. (2010). HIV Neutralizing Antibodies: Clinical Correlates and Implications for Vaccines. J. Infect. Dis..

[44] Plotkin S.A., Plotkin S.A. (2008). Correlates of Vaccine-Induced Immunity. Clin. Infect. Dis..

[45] Plotkin S.A. (2023). Recent Updates on Correlates of Vaccine-Induced Protection. Front. Immunol..

[46] Francis T., Korns R.F., Voight R.B., Boisen M., Hemphill F.M., Napier J.A., Tolchinsky E. (1955). An Evaluation of the 1954 Poliomyelitis Vaccine Trials. Am. J. Public Health Nations Health.

[47] Gilbert P.B. (2010). Some Design Issues in Phase 2B vs. Phase 3 Prevention Trials for Testing Efficacy of Products or Concepts. Stat. Med..

[48] Carlson N. (2010). Phase 2B Clinical Trials—A Useful Interim Step to a Phase 3 Clinical Trial. Sci. Transl. Med..

[49] Plotkin S.A., Gilbert P.B. (2012). Nomenclature for Immune Correlates of Protection After Vaccination. Clin. Infect. Dis..

[50] Gilbert P.B., Fong Y., Kenny A., Carone M. (2022). A Controlled Effects Approach to Assessing Immune Correlates of Protection. Biostatistics.

[51] Gilbert P.B., Donis R.O., Koup R.A., Fong Y., Plotkin S.A., Follmann D. (2022). A Covid-19 Milestone Attained—A Correlate of Protection for Vaccines. N. Engl. J. Med..

[52] Heng F., Magaret C.A., Rouphael N.G., Branche A.R., Fong Y., Carpp L.N., Yu C., Chen S., Zhang B., Diemert D.J. (2026). The Neutralizing Antibody Titer Correlate of COVID-19 Risk in the COVID-19 Variant Immunologic Landscape (COVAIL) Trial Was Not Modified by SARS-CoV-2 Amino Acid Sequence Distances. Vaccine.

[53] Corey L., Gilbert P.B., Tomaras G.D., Haynes B.F., Pantaleo G., Fauci A.S. (2015). Immune Correlates of Vaccine Protection against HIV-1 Acquisition. Sci. Transl. Med..

[54] Gray G., Buchbinder S., Duerr A. (2010). Overview of STEP and Phambili Trial Results: Two Phase IIb Test-of-Concept Studies Investigating the Efficacy of MRK Adenovirus Type 5 Gag/Pol/Nef Subtype B HIV Vaccine. Curr. Opin. HIV AIDS.

[55] Collins R., Bowman L., Landray M., Peto R. (2020). The Magic of Randomization versus the Myth of Real-World Evidence. N. Engl. J. Med..

[56] Stephenson K.E., Marcelin J.R., Pettifor A.E., Janes H., Brown E., Neradilek M., Yen C., Andriesen J., Grunenberg N., Espy N. (2023). Efficacy of Messenger RNA–1273 Against Severe Acute Respiratory Syndrome Coronavirus 2 Acquisition in Young Adults From March to December 2021. Open Forum Infect. Dis..

[57] Graybill L.A., Chi B.H. (2023). PrEParing for Choice in a New Era of HIV Prevention. Lancet HIV.

[58] Bekker L., Pike C., Hillier S.L. (2022). HIV Prevention: Better Choice for Better Coverage. J. Int. AIDS Soc..

[59] Gilbert P.B., Janes H.E., Huang Y. (2016). Power/Sample Size Calculations for Assessing Correlates of Risk in Clinical Efficacy Trials. Stat. Med..

[60] Fong Y., Huang Y., Benkeser D., Carpp L.N., Áñez G., Woo W., McGarry A., Dunkle L.M., Cho I., Houchens C.R. (2023). Immune Correlates Analysis of the PREVENT-19 COVID-19 Vaccine Efficacy Clinical Trial. Nat. Commun..

[61] Janes H.E., Cohen K.W., Frahm N., Rosa S.C.D., Sanchez B., Hural J., Magaret C.A., Karuna S., Bentley C., Gottardo R. (2017). Higher T-Cell Responses Induced by DNA/rAd5 HIV-1 Preventive Vaccine Are Associated with Lower HIV-1 Infection Risk in an Efficacy Trial. J. Infect. Dis..

[62] Beyrer C., Tomaras G.D., Gelderblom H.C., Gray G.E., Janes H.E., Bekker L.-G., Millett G., Pantaleo G., Buchbinder S., Corey L. (2024). Is HIV Epidemic Control by 2030 Realistic?. Lancet HIV.

[63] Gray G.E., Mngadi K., Lavreys L., Nijs S., Gilbert P.B., Hural J., Hyrien O., Juraska M., Luedtke A., Mann P. (2024). Mosaic HIV-1 Vaccine Regimen in Southern African Women (Imbokodo/HVTN 705/HPX2008): A Randomised, Double-Blind, Placebo-Controlled, Phase 2b Trial. Lancet Infect. Dis..

[64] Sadoff J., Gray G., Vandebosch A., Cárdenas V., Shukarev G., Grinsztejn B., Goepfert P.A., Truyers C., Dromme I.V., Spiessens B. (2022). Final Analysis of Efficacy and Safety of Single-Dose Ad26.COV2.S. N. Engl. J. Med..

[65] Goga A.E., Bekker L.-G., Garrett N., Takuva S., Sanne I., Odhiambo J., Mayat F., Fairall L., Brey Z., Bamford L. (2021). Sisonke Phase 3B Open-Label Study: Lessons Learnt for National and Global Vaccination Scale-up during Epidemics. S. Afr. Med. J..

[66] Bekker L.-G., Garrett N., Goga A., Fairall L., Reddy T., Yende-Zuma N., Kassanjee R., Collie S., Sanne I., Boulle A. (2022). Effectiveness of the Ad26.COV2.S Vaccine in Health-Care Workers in South Africa (the Sisonke Study): Results from a Single-Arm, Open-Label, Phase 3B, Implementation Study. Lancet.

[67] Shinde V., Bhikha S., Hoosain Z., Archary M., Bhorat Q., Fairlie L., Lalloo U., Masilela M.S.L., Moodley D., Hanley S. (2021). Efficacy of NVX-CoV2373 Covid-19 Vaccine against the B.1.351 Variant. N. Engl. J. Med..

[68] Garrett N., Tapley A., Hudson A., Dadabhai S., Zhang B., Mgodi N.M., Andriesen J., Takalani A., Fisher L.H., Kee J.J. (2025). Hybrid versus Vaccine Immunity of mRNA-1273 among People Living with HIV in East and Southern Africa: A Prospective Cohort Analysis from the Multicentre CoVPN 3008 (Ubuntu) Study. eClinicalMedicine.

[69] Aiyegbusi O.L., Davies E.H., Myles P., Williams T., Frost C., Haroon S., Hughes S.E., Wilson R., McMullan C., Subramanian A. (2023). Digitally Enabled Decentralised Research: Opportunities to Improve the Efficiency of Clinical Trials and Observational Studies. BMJ Evid. Based Med..

[70] Miyata B.L., Tafuto B., Jose N. (2023). Methods and Perceptions of Success for Patient Recruitment in Decentralized Clinical Studies. J. Clin. Transl. Sci..

[71] WHO WHO Expands Recommendation on Oral Pre-Exposure Prophylaxis of HIV Infection (PreP). https://iris.who.int/bitstream/handle/10665/197906/WHO_HIV_2015.48_eng.pdf.

[72] Johnson L.F., van Schalkwyk C., Dorrington R.E. Modelling the Impact of HIV in South Africa’s Provinces: 2025 Update. https://thembisa.org/content/downloadPage/Province2025.

[73] Martin C.E., Ramatsoma H., Chidumwa G., Nongena P., Letsielo M.J., Kutywayo A., Koloane N., Ncube S., Mdluli N., Nattey C. (2026). Uptake of Dapivirine Vaginal Ring and Oral Pre-Exposure Prophylaxis among Women Accessing HIV Prevention Services in South Africa: A Cohort Study. Lancet HIV.

[74] Fonner V.A., Irungu E., Conlon M., Akello C.A., Gwavava E., K’Orimba K., Naidoo N.P., Jeckonia P., Mahaka I., Mullick S. (2025). PrEP Choice in the Real World: Results of a Prospective Cohort Study Describing Uptake and Use Patterns of Oral PrEP and the Dapivirine Vaginal Ring among Women in sub-Saharan Africa. J. Int. AIDS Soc..

[75] Shangase N., Jiyane A., Buthelezi F., O’Connor C., Brown B., Rees K. (2025). Pre-Exposure Prophylaxis Persistence at Two Sites in an Integrated Primary Health Care Programme in South Africa. Front. Public Health.

[76] Rao A., Mhlophe H., Comins C., Young K., Mcingana M., Lesko C., Mulumba N., Baral S., Hausler H., Schwartz S. (2022). Persistence on Oral Pre-Exposure Prophylaxis (PrEP) among Female Sex Workers in eThekwini, South Africa, 2016–2020. PLoS ONE.

[77] Celum C.L., Bukusi E.A., Bekker L.G., Delany-Moretlwe S., Kidoguchi L., Omollo V., Rousseau E., Travill D., Morton J.F., Mogaka F. (2022). PrEP Use and HIV Seroconversion Rates in Adolescent Girls and Young Women from Kenya and South Africa: The POWER Demonstration Project. J. Int. AIDS Soc..

[78] Admassu M., Nöstlinger C., Hensen B. (2024). Barriers to PrEP Use and Adherence among Adolescent Girls and Young Women in Eastern, Southern, and Western Africa: A Scoping Review. BMC Women’s Health.

[79] Mongwenyana-Makhutle C., Hendrickson C., Dubazana S., Mazibuko J.M., Motaung R., Mokhesi N., Bokolo S., Chetty-Makkan C., Moolla A., Long L. (2026). “Taking PrEP Every Day for Me Is a Challenge”: Barriers, and Facilitators to Accessing HIV Pre-Exposure Prophylaxis (PrEP) Services among Young People in Gauteng, South Africa. medRxiv.

[80] Landovitz R.J., Nguyen L. (2025). Severe Lenacapavir Injection Site Nodule. AIDS.

[81] Mann C.Z., Sales A.C., Gagnon-Bartsch J.A. (2025). Combining Observational and Experimental Data for Causal Inference Considering Data Privacy. J. Causal Inference.

[82] Karlsson R., Wang G., Bartolomeis P.D., Krijthe J.H., Dahabreh I.J. (2024). Robust Integration of External Control Data in Randomized Trials. arXiv.

[83] Bannick M., Donnell D., Hayes R., Laeyendecker O., Gao F. (2024). An Enhanced Cross-sectional HIV Incidence Estimator That Incorporates Prior HIV Test Results. Stat. Med..

[84] Facente S.N., Grebe E., Maher A.D., Fox D., Scheer S., Mahy M., Dalal S., Lowrance D., Marsh K. (2022). Use of HIV Recency Assays for HIV Incidence Estimation and Other Surveillance Use Cases: Systematic Review. JMIR Public Health Surveill..

[85] Gao F., Bannick M. (2022). Statistical Considerations for Cross-sectional HIV Incidence Estimation Based on Recency Test. Stat. Med..

[86] Oyo-Ita A., Oduwole O., Arikpo D., Effa E.E., Esu E.B., Balakrishna Y., Chibuzor M.T., Oringanje C.M., Nwachukwu C.E., Wiysonge C.S. (2023). Interventions for Improving Coverage of Childhood Immunisation in Low- and Middle-income Countries. Cochrane Database Syst. Rev..

[87] WHO Reaching Every District (RED). https://www.afro.who.int/sites/default/files/2018-01/Reaching%20Every%20District%20(RED)%20English%20F%20web%20v2.pdf.

[88] Ouédraogo H.S., Kabore Y.L.B., Sawadogo A.G., Bakouan M., Sawadogo N., Mano M., Zongo A., Sanou S., Kaboré L. (2023). Task-Shifting Immunization Activities to Community Health Workers: A Mixed-Method Cross-Sectional Study in Sahel Region, Burkina Faso. Glob. Health Sci. Pract..

[89] Gsell P.-S., Camacho A., Kucharski A.J., Watson C.H., Bagayoko A., Nadlaou S.D., Dean N.E., Diallo A., Diallo A., Honora D.A. (2017). Ring Vaccination with rVSV-ZEBOV under Expanded Access in Response to an Outbreak of Ebola Virus Disease in Guinea, 2016: An Operational and Vaccine Safety Report. Lancet Infect. Dis..

[90] Elliott T., Bradshaw D., Fidler S. (2025). HIV Diagnosis during Acute Infection: Implications of Long-Acting Preexposure Prophylaxis and Other Evolving Challenges. Curr. Opin. HIV AIDS.

[91] Zwiers L.C., Veen D., Mitratza M., Brakenhoff T.B., Goodale B.M., Klaver P., Hage K.Y., van Willigen M., Downward G.S., Lugtig P. (2025). Increasing Retention in a Large-Scale Decentralized Clinical Trial: Learnings From the COVID-RED Trial. Mayo Clin. Proc. Digit. Health.

[92] Cussen A., Littler K. (2025). Trial Characteristics, Methods and Reported Challenges of Decentralised Clinical Trials: A Scoping Review. BMJ Open.

[93] AVAC Good Participatory Practice: Guidelines for Biomedical HIV Prevention Trials, Second Edition. https://avac.org/resource/report/good-participatory-practice-guidelines-for-biomedical-hiv-prevention-trials-second-edition/.

[94] MacQueen K.M., Bhan A., Frohlich J., Holzer J., Sugarman J., Ethics Working Group of the HIV Prevention Trials Network (2015). Evaluating Community Engagement in Global Health Research: The Need for Metrics. BMC Med. Ethics.

[95] Hammer S.M., Sobieszczyk M.E., Janes H., Karuna S.T., Mulligan M.J., Grove D., Koblin B.A., Buchbinder S.P., Keefer M.C., Tomaras G.D. (2013). Efficacy Trial of a DNA/rAd5 HIV-1 Preventive Vaccine. N. Engl. J. Med..

[96] Blease C.R., Bishop F.L., Kaptchuk T.J. (2017). Informed Consent and Clinical Trials: Where Is the Placebo Effect?. BMJ.

[97] Caille A., Billot L., Kasza J. (2024). Practical and Methodological Challenges When Conducting a Cluster Randomized Trial: Examples and Recommendations. J. Epidemiol. Popul. Health.

[98] Buchbinder S.P., Guzman S.S., Sanchez J., Willems W., Stieh D.J., van Duijn J., van Rosmalen M.G.M., Hendriks J., Nijs S., Lavreys L. (2025). Efficacy and Safety of a Mosaic HIV-1 Vaccine Regimen in Men Who Have Sex with Men and Transgender Individuals (HVTN 706/HPX3002/Mosaico): A Global, Randomised, Double-Blind, Placebo-Controlled, Phase 3 Trial. Lancet HIV.

[99] Masignani V., Palacios R., Martin M.-T., Tibaldi F., Vadivelu V.K. (2025). De-Risking Vaccine Development: Lessons, Challenges, and Prospects. npj Vaccines.

